# Relationship between Watershed Infarcts and Recent Intra Plaque Haemorrhage in Carotid Atherosclerotic Plaque

**DOI:** 10.1371/journal.pone.0108712

**Published:** 2014-10-01

**Authors:** Clothilde Isabel, Augustin Lecler, Guillaume Turc, Olivier Naggara, Emmanuelle Schmitt, Samia Belkacem, Catherine Oppenheim, Emmanuel Touzé

**Affiliations:** 1 Université Paris Descartes, Service de Neurologie, Inserm U894, Paris, France; 2 Université Paris Descartes, Service de Neuroradiologie, Inserm U894, Paris, France; 3 Service de Neurologie, CHRU Nancy, Nancy, France; 4 Service de neurologie, Groupe Hospitalier Pitié-Salpêtrière, Paris, France; Medical University of Graz, Austria

## Abstract

**Objective:**

Watershed infarcts (WSI) are thought to result from hemodynamic mechanism, but studies have suggested that microemboli from unstable carotid plaques may distribute preferentially in watershed areas, *i.e.,* between two cerebral arterial territories. Intraplaque haemorrhage (IPH) is an emerging marker of plaque instability and microembolic activity. We assessed the association between WSI and IPH in patients with recently symptomatic moderate carotid stenosis.

**Methods and Results:**

We selected 65 patients with symptomatic moderate (median NASCET degree of stenosis = 31%) carotid stenosis and brain infarct on Diffusion-Weighted Imaging (DWI) on Magnetic Resonance Imaging (MRI) from a multicentre prospective study. Fourteen (22%) had WSI (cortical, n = 8; internal, n = 4; cortical and internal, n = 2). Patients with WSI were more likely to have IPH than those without WSI although the difference was not significant (50% *vs.* 31%, OR = 2.19; 95% CI, 0.66–7.29; *P* = 0.20). After adjustment for degree of stenosis, age and gender, the results remained unchanged.

**Conclusion:**

About one in fifth of brain infarcts occurring in patients with moderate carotid stenosis were distributed in watershed areas. Albeit not significant, an association between IPH - more generally plaque component - and WSI, still remains possible.

## Introduction

Watershed infarcts (WSI) involve the junction of the distal fields of two non-anastomosing arterial systems. They represent approximately 10% of all ischemic strokes [1], but imaging studies in patients with internal carotid artery (ICA) stenosis report an incidence ranging from 19% to 64% [2]. The vulnerability of distal watershed (WS) areas to ischemia is thought to result from low perfusion. However, despite numerous studies dedicated to WSI [2], the pathogenesis of WSI remains debated. The most consensual mechanism is hemodynamic failure due to hypotension in the presence of severe ICA disease [3]–[5]. However, several experimental and human studies [6]–[8] have found an association between WSI and microemboli arising from unstable carotid plaques [1], [2], [9], from the stump of an occluded ICA, or even in patients with hypereosinophilic syndromes [10], suggesting that small thrombi travel preferentially towards WS areas. However, clinical studies supporting this hypothesis remain scarce.

Carotid intraplaque haemorrhage (IPH) is a marker of plaque instability [11]. It has been shown to be associated with symptomatic carotid disease, spontaneous microembolic activity at transcranial Doppler, and with recurrent risk of ischemic stroke [12]–[14]. We hypothesized that if micro thrombi from unstable carotid plaque tend to embolize preferentially to WS areas, then IPH should be more common in patients with WSI. We therefore assessed the association between WSI and IPH in patients with recently symptomatic moderate carotid stenosis from the HIgh-Resolution magnetic resonance Imaging in atherosclerotic Stenosis of the Carotid artery (HIRISC) study.

## Methods

### Population

HIgh-Resolution magnetic resonance Imaging in atherosclerotic Stenosis of the Carotid artery (HIRISC) is an ongoing multicenter prospective study assessing the prognostic value of carotid plaque vulnerability, as defined on MRI [15]. Recruitment started in October 2003, and all except 1 center stopped recruiting patients in May 2009. Patients were identified among inpatients and outpatients by local investigators and were eligible for the study if they (1) had symptomatic (≥50% according to European Carotid Surgery Trial (ECST) and <70% according to North American Symptomatic Carotid Endarterectomy Trial (NASCET) method) or asymptomatic (≥50% according to NASCET) stenosis of the internal carotid artery (ICA); (2) were not scheduled for endarterectomy within the next 6 months; and (3) did not have any other major cause of stroke. As we hypothesized that patients with large symptomatic plaque associated with a relatively low degree of NASCET stenosis (i.e., mainly detected by the ECST method) have a relatively high risk of recurrent event, we used the ECST method to select patients, whether or not resulting in NASCET stenosis. For the present analysis, we selected all patients with moderate (non haemodynamic) symptomatic carotid stenosis and proven brain infarct on MRI from 3 centres, *i.e.* those in which brain MRI was used to confirm the diagnosis of ischemic stroke. The local ethics committee (Comité Consultatif de Protection des Personnes dans la Recherche Biomedicale, CCPPRB Paris-Cochin) approved the study and all patients signed an informed consent.

### Carotid plaque imaging

All patients were imaged on 1.5-T magnetic resonance units using the same 4-channel phased-array surface coil (Machnet), including 4 pulse sequences: 3-dimensional time of flight, T1-weighted, proton density-weighted, and T2-weighted, as detailed elsewhere [15].

Two independent readers blinded to clinical data examined plaque MRI images as previously reported [15]. Recent IPH was defined as hyperintensity on all 4-pulse sequences on ≥1 slice, the reference being the signal of the adjacent sternocleidomastoid muscle. The location of the IPH was classified as juxtaluminal, intermediate, adventitial, or total. Cases of disagreement between readers were solved by consensus. Area measurements were obtained for each location by manually tracing the boundaries of each component. We also calculated the ratio of IPH to wall (i.e., vessel minus lumen) areas for each patient, which we categorized as <40% or ≥40% (corresponding to the median value). Finally, we analysed the association with cap rupture. Fibrous cap was defined as a hypersignal band between the lumen and the lipid rich/necrotic core area on T2-weighted sequence.

### Diagnostic and definition of WSI

**Figure 1:**
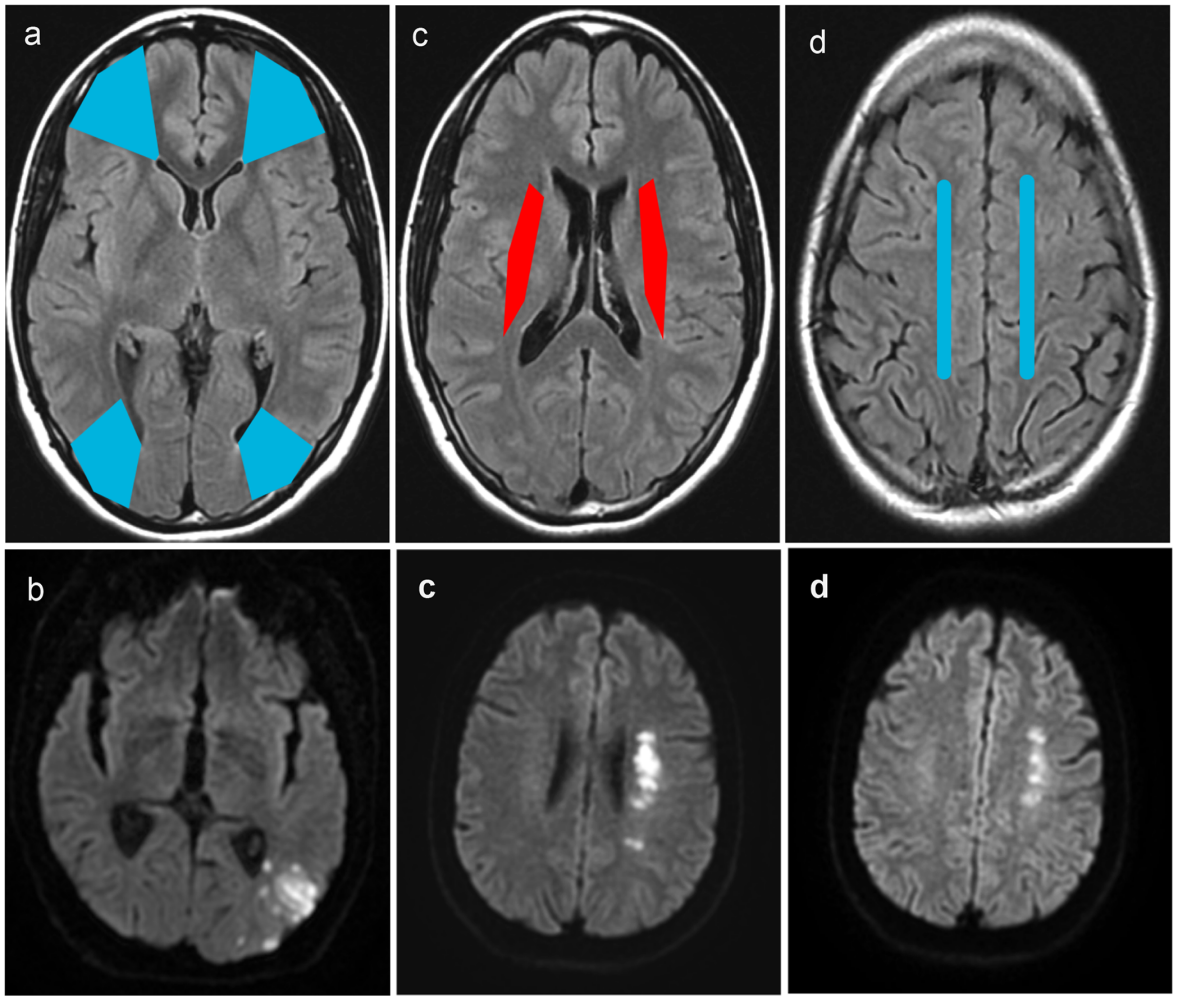
Watershed territories, on sequence Fluid Attenuated Inversion Recovery (FLAIR; at the top) and corresponding illustrative cases Diffusion-Weighted Imaging (DWI; below); in blue: cortical watershed, in red: internal watershed; (a) frontal WSI: between the anterior and the middle cerebral arterial territories, (a, b) occipital WSI: at the boundaries of the middle and the posterior arterial territories; (c) internal WSI: between territories of superficial and deep perforators of the middle cerebral artery; (d) paramedian WSI: between the anterior and the middle cerebral arterial territories. WSI = watershed infarct.

Diffusion Weighted Imaging (DWI) MRI was done in all patients within the first 24 hours after admission. DWI was independently reviewed by two readers in patients with established infarct (hypersignal on this sequence), blinded to plaque imaging data. Cases of disagreement between readers were solved by consensus and, where necessary, by a third expert reader. WSI were defined as suggested in previous publications [2], [11], and classified as cortical or internal (Figure 1).

### Statistical Analysis

Variables were compared using the *t*-test, Pearson chi-2 test, or Fischer exact test, as appropriate. Association between WSI and recent IPH was assessed by calculation of Odds Ratios (ORs) through uni- and multivariate logistic regression models.

## Results

### Population

Among the 258 patients included in the HIRISC study, 210 were recruited from the 3 centres in which MRI was used as first line imaging for the diagnosis of ischemic stroke. 105 patients had symptomatic stenosis. Among those, 35 had no ischemic brain lesion on MRI (31 Transient Ischemic Attacks, 4 retinal infarcts) and 70 patients had brain infarct proven on DWI MRI. Five patients were excluded because no plaque MRI was performed (claustrophobia, n = 4; death before MRI being done, n = 1), leaving 65 patients for the present analysis. Their mean age was 67.4 years (range, 40–88) and the median (IQR) degree of stenosis was 31% (17,5–41) according to NASCET. There were 17 women and 48 men.

### IPH and WSI

Among the 65 patients, 14 (22%) had WSI (cortical, n = 8; internal, n = 4; cortical and internal, n = 2). Among the cortical WSI, one was frontal (associated with internal WSI), one paramedian, 4 were occipital (of which one with internal WSI) and 4 affected two watershed areas (one frontal and occipital, 3 paramedian and occipital). As shown in table 1, patients with WSI did not significantly differ from those without WSI regarding age or risk factors. Overall, 23 (35%) patients had IPH on plaque MRI (median [IQR] volume = 17.7 [10–21.6] mm^3^). Patients with WSI were more likely to have IPH than those without WSI although the difference was not statistically significant (50% vs. 31%, OR = 2.19; 95% CI, 0.66–7.29; P = 0.20). After adjustment for degree of stenosis, age and gender, the relation was unchanged (OR = 2.17; 0.64–7.31). The results were similar when patients with pure internal WSI (OR = 2.24; 0.54–9.27) or with pure external WSI were excluded (OR = 4.72; 0.74–30.19). Looking at patients with IPH visible on more than two-3 mm thick slices (ie large IPH) or at patients with an IPH-to-wall ratio ≥40%, results were similar (data not shown). Finally, we observed a nonsignificant association between WSI and cap rupture on T2 (OR = 3.80; 0.66–21.77).

**Table 1:** Comparison between patients with watershed infarct (WSI +) and those with no watershed infarct (WSI -).

	WSI+(n = 14)	WSI - (n = 51)
**Patients Characteristics**		
Age, median (IQR), years	67 (60–71)	69 (59–75)
Male gender, n (%)	11 (79)	37 (73)
High blood pressure, n (%)	8 (57)	33 (65)
Diabetes, n (%)	3 (21)	8 (16)
Dyslipemia, n (%)	9 (64)	28 (55)
Current smoking, n (%)*	5 (36)	14 (28)
**Carotid disease**		
Median degree of stenosis (interquartile range), %		
NASCET	40 (25–50)	30 (15–39)
ECST	60 (52–70)	60 (52–65)
**Recent IPH**		
Presence of IPH (≥1 slice), n (%)	7 (50%)	16 (31%)

*one data missing.

## Discussion

Hypothesizing that microemboli from unstable carotid plaque should distribute preferentially to WS area, we found that about one in fifth of brain infarcts occurring in patients with nonhemodynamic carotid stenosis were distributed in WS areas. Although, to the best of our knowledge, it is the first study that explores the association between recent IPH and WSI, we failed to demonstrate a statistically significant link. However, such an association is not excluded since the odds ratio was 2.2 and our statistical power was only 60% to detect an odds ratio of 2.

The pathogenesis of WSI remains debated. In patients with carotid stenosis, hypoperfusion is thought to cause WSI only under the circumstance of cardiac arrest or severe systemic hypoperfusion [4], [5]. Therefore, the vast majority of strokes distal to a carotid stenosis result from artery-to-artery embolism. In fact, autopsy and imaging studies have shown that, after cardiac surgery or after embolization from the aorta, brain infarcts tend to be distributed bilaterally within the brain WS regions, suggesting that borderzone areas are more prone to be involved in case of embolism [6], [7], [16]. Some authors have hypothesized that emboli that enter the vascular bed of distal or WS regions are less likely to be washed-out and cleared [16]. This prevailing distribution in WS areas has been demonstrated mainly in patients with severe carotid stenosis, suggesting that low perfusion is an important determinant in clearance of emboli. However, in such patients, it may be challenging to identify the respective contribution of hypoperfusion and embolization, as both likely act together in the genesis of WSI. Our population was particularly interesting because all patients had moderate stenosis *i.e.*, none had severe haemodynamic impairment. The finding that about 20% of brain infarcts occurring in patients with moderate carotid stenosis were distributed in WS areas is in agreement with the hypothesis that WS areas are susceptible to ischemia.

IPH has been shown to be associated with plaque vulnerability and a high risk of stroke in cross-sectional [12], [14] and longitudinal studies [17]. Previous studies have shown that ulcerated or heterogeneous plaque can produce spontaneous micro-embolic activity at transcranial Doppler [2], [18]. Regarding IPH, only one study has suggested a link between IPH and micro-emboli, although the relation was not statistically significant [13]. This plaque feature is quite easy to identify on MRI with an excellent intra- and inter-observe reproducibility [15]. IPH is therefore among the most promising imaging risk factor to identify high-risk patients. However, there are probably other important plaque characteristics that are associated with plaque vulnerability. Although we also failed to show a significant association between WSI and cap rupture, we cannot exclude such an association considering the poor reproducibility of cap analysis on MRI, especially in case of IPH [15].

Emboli distribution does not depend only on plaque vulnerability. There are several other potential important factors that could influence the distribution of emboli from atherosclerotic lesions in the brain arteries, such as size and thrombus composition. In an experimental study, particles in the 150–210 µm size range distributed preferentially to the watershed zone whereas particles less than 150 µm in size were randomly dispersed in the leptomeningeal arteries of all vascular regions [8]. Otherwise, autopsy studies have shown that composition of the thrombi differs according to the affected territory: occluding emboli in the watershed regions mostly are made of cholesterol crystals with little fibrin whereas those in the core of the arterial territory contain cholesterol crystals and more fibrin [6].

Although we have previously shown almost perfect intraobserver (κ = 0.82; 95% CI 0.68–0.96) and substantial interobserver (κ = 0.62; 95% CI 0.43–0.81) agreements for the identification of IPH on MRI [15], our study has several limitations. First, although we used a large prospective cohort of patients with carotid disease, only half were symptomatic and not all had brain MRI at inclusion. The statistical power was low and our findings are to be interpreted cautiously. Second, the range of arterial stenosis was too narrow to seek for a potential interaction with hemodynamic impairment and the relation between IPH and WSI. Third, we cannot exclude misclassification in WSI diagnosis, although we had two independent readers, consensus assessment and third expert reader in case of disagreement.

In conclusion, about one in fifth of brain infarcts occurring in patients with moderate carotid stenosis were distributed in watershed areas. Albeit not significant, an association between IPH - more generally plaque components - and WSI, cannot be excluded. More studies need to be developed to improve the management of patients with nonhemodynamic carotid stenosis, according to the composition of the plaque on MRI.
